# Microplastic Pollution in the Environment: A Chemical Engineering Perspective on Sources, Fate, and Mitigation Strategies

**DOI:** 10.3390/polym18010029

**Published:** 2025-12-23

**Authors:** Mahmoud Allawy Mohsin, Ahmed Hayder Abd zaid

**Affiliations:** College of Arts and Sciences, American University of Iraq-Baghdad, Airport Road, Baghdad 10023, Iraq; ahmed.abdzaid@auib.edu.iq

**Keywords:** microplastics, techno-economic analysis (TEA), advanced adsorbents, wastewater treatment, adsorption, circular economy, biodegradable polymers

## Abstract

Microplastic pollution is a defining environmental crisis of the Anthropocene, threatening ecosystems and human health due to its persistence and global dispersion. This review synthesizes current knowledge through a chemical engineering framework, analyzing the contaminant’s lifecycle from formation and environmental fate to detection and removal. We systematically evaluate conventional and advanced mitigation technologies, highlighting the potential of engineered adsorbents (e.g., functionalized sponges, biochar) for targeted capture while underscoring the limitations of current wastewater treatment for nano-plastics. The analysis extends beyond end-of-pipe solutions to underscore the imperative for sustainable polymer design and circular economy systems, where biodegradable polymers and chemical recycling must be integrated. Crucially, we identify techno-economic analysis (TEA) and life-cycle assessment (LCA) as essential, yet underdeveloped, tools for quantifying the true cost and sustainability of management strategies. The synthesis concludes that addressing microplastic pollution requires the integrated application of chemical engineering principles across molecular, process, and system scales, and it identifies key research priorities in advanced material design, standardized analytics, hybrid treatment processes, and comprehensive impact modeling.

## 1. Introduction

The pervasive and persistent contamination of global ecosystems by microplastics (MPs) represents a defining environmental challenge of the Anthropocene, gathering significant scientific and public concern due to their widespread distribution and potential negative impacts on ecological function and human health. Defined as synthetic solid particles or polymeric matrices with dimensions ranging from 1 µm to 5 mm, MPs originate from a multitude of sources and persist in the environment for extended periods due to the recalcitrant nature of many synthetic polymers [1]. The very properties that make them persistent pollutants their small size and chemical stability also make their detection and removal exceptionally difficult, presenting a complex challenge that demands the systematic, scalable solutions offered by chemical engineering principles. Microplastics are broadly classified into two categories based on their origin: primary and secondary, though definitions can vary within the literature [1]. The most common distinction is based on the point of release into the environment. Primary microplastics are those that enter the environment already as microscopic particles. This category includes intentionally manufactured microbeads (e.g., from personal care products) and industrial plastic pellets (nurdles). Under this release-based framework, it also encompasses wear particles such as microfibers shed from synthetic textiles during washing and tire wear particles generated from road abrasion, as these are emitted directly as microplastics during a product’s use phase [1,2]. Secondary microplastics result from the environmental fragmentation and degradation of larger plastic debris (macro-plastics) due to ultraviolet radiation, mechanical abrasion, and biological weathering. Common sources include mismanaged plastic waste like single-use bags, bottles, and abandoned fishing gear [1,2]. Some alternative classifications focus on the intent of production, considering only deliberately manufactured micro-sized items as primary. For clarity and consistency, this review adopts the prevalent release-based framework, recognizing wear particles as a major primary source. The complexity of the microplastic problem is underscored by the diversity of polymer types present in the environment. The “top five” most frequently encountered plastics are: polyethylene (PE), polypropylene (PP), polystyrene (PS), polyethylene terephthalate (PET), and polyvinyl chloride (PVC), which reflect their extensive use in packaging, construction, and consumer goods [3]. The environmental fate and transport of these polymers are governed by their inherent physicochemical properties such as density, hydrophobicity, and surface charge as summarized in Table 1. For instance, a polymer’s density determines its initial buoyancy, while its surface chemistry influences interactions with other particles, pollutants, and biological surfaces.

For instance, the density of these polymers is a primary factor governing their initial distribution in aquatic systems. Low-density polymers like polypropylene (PP, ~0.90–0.92 g/cm^3^) and low-density polyethylene (LDPE, ~0.91–0.94 g/cm^3^) are positively buoyant and tend to float. Polymers with densities significantly greater than water, such as polyethylene terephthalate (PET, ~1.38 g/cm^3^) and polyvinyl chloride (PVC, ~1.3–1.45 g/cm^3^), readily sink. High-density polyethylene (HDPE, ~0.94–0.97 g/cm^3^) presents an intermediate case; with a density much closer to that of water than LDPE, it displays near-neutral buoyancy. Its vertical transport is therefore not predetermined by density alone but is dynamically controlled by environmental processes such as biofilm growth (biofouling) and aggregation, which can alter its effective density and facilitate movement between the water column and sediments. Furthermore, their chemical structures and surface properties, such as the slight negative surface charge most exhibit in aqueous environments, dictate their susceptibility to degradation, interaction with co-contaminants, and potential for bioavailability to organisms [3]. This review aims to synthesize the current scientific and engineering knowledge on microplastic pollution through the unifying framework of chemical engineering. While existing literature often treats the topics of detection, fate, and mitigation in isolation, this work provides a comprehensive and integrated analysis that links fundamental material properties to scalable environmental solutions. To this end, the review has the following specific objectives: (i) To analyze the sources, environmental transformations, and fate of microplastics through the lens of chemical reaction engineering and transport phenomena. (ii) To evaluate engineered mitigation and removal technologies from conventional unit operations to advanced materials with a focus on process efficiency, scalability, and techno-economic viability. (iii) To apply systems-level analysis tools, including techno-economic analysis (TEA) and life-cycle assessment (LCA), to assess the sustainability of management strategies and the transition towards a circular plastic economy. (iv) To identify critical research gaps and future directions at the intersection of materials science, process engineering, and environmental systems analysis.

The review applies a chemical engineering framework to integrate scientific and engineering knowledge on microplastic pollution. It systematically proceeds from the fundamental science (formation and fate) to engineered solutions (detection and mitigation), concluding with a techno-economic and life-cycle analysis of these approaches.

## 2. Sources, Formation Mechanisms, and Polymer Fragmentation

Building upon the fundamental classification of microplastics, their continuous entry into and proliferation within the environment is driven by a complex array of sources and transformation pathways. As established, microplastics (MPs) are broadly categorized into primary and secondary types, a distinction critical for developing targeted mitigation strategies [4].

### 2.1. Primary Microplastics

Primary microplastics are deliberately manufactured as microscopic particles and are directly released into the environment. These particles originate from a diverse range of diffuse and point sources, often linked to everyday products and industrial processes.

A significant contributor is the wear and tear of synthetic textiles. Clothing made from materials like polyethylene terephthalate (PET) sheds thousands of microfibers during a single washing cycle, constituting a major source of aquatic microplastic pollution [5]. Similarly, vehicle tire wear represents a substantial, yet often overlooked, source. Modern tires contain significant amounts of synthetic polymers, and their abrasion against road surfaces releases particles whose emission rate is influenced by tire composition, vehicle dynamics, and road texture. The increasing weight of electric vehicles is anticipated to further exacerbate this emission pathway [6]. Abrasion from road markings, which contain synthetic resins, is also identified as a notable contributor, with estimates suggesting they could be responsible for up to 7% of total microplastic emissions [7].

Other significant sources include marine coatings (e.g., polyurethane, epoxy, vinyl) that release particles through weathering and scraping during vessel maintenance [8], and city dust, an aggregate category encompassing microplastics from artificial turf, building paints, industrial abrasives, and the complex lifecycle of tire wear particles (TWPs) as they are deposited on roads, emitted into the air, or washed into sewers [9]. In agricultural settings, primary microplastics are introduced via polymer-coated slow-release fertilizers and plastic mulch films, which have been identified as key sources of soil contamination [10]. Finally, a wide array of other industrial and consumer products, including drilling fluids, industrial abrasives, detergent additives, and plastic media used in wastewater treatment, contribute to this diverse emission profile [11].

The diverse origins and pathways of microplastic release into the environment are complex. To provide a clear overview, the primary categories of microplastic sources, their common polymer types, and dominant release mechanisms are summarized in Table 2.

### 2.2. Secondary Microplastics

Secondary microplastics are not deliberately produced but originate from environmental degradation and fragmentation of larger plastic items. This process continuously generates microplastic pollution from the vast reservoir of macroscopic plastic waste, which includes abundant items like beverage bottles, food containers, single-use cutlery, carry bags, fishing gear, and a multitude of other consumer and industrial products [14].

The degradation of larger plastic debris is initiated by exposure to environmental agents such as solar ultraviolet (UV) radiation, heat, wind, wave action, and physical abrasion, which collectively embrittle and break down objects like bags, bottles, and fishing nets [12]. Notably, some plastics are designed to fragment more readily; oxo-degradable plastics, which contain additives to accelerate their breakdown under UV light, are a concerning source of secondary microplastics and are now facing regulatory bans in regions like the EU [15]. Beyond litter, the general wear and tear of everyday plastic items, from furniture and toys to household goods, continuously shed microplastic particles [16]. A particularly persistent source in marine environments is discarded fishing gear and shipping waste, such as “ghost nets,” which undergo prolonged weathering and abrasion, steadily releasing microplastic fragments into the ocean [13].

### 2.3. Mechanisms of Fragmentation and Degradation

The degradation of plastic debris into micro- and nano-plastics is a complex process governed by the interplay of abiotic (physical and chemical) and biotic (biological) mechanisms, which often act synergistically to accelerate polymer breakdown [1,17]. This transformation has critical implications for the environmental fate, transport, and toxicity of plastic particles [18,19].

**Figure 1:**
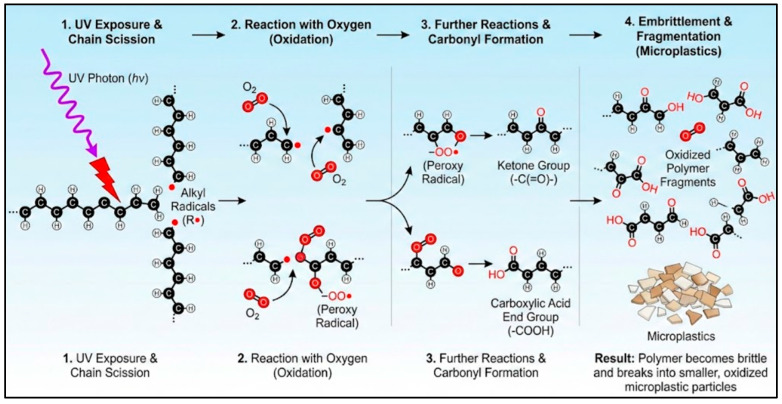
Schematic representation of UV-driven photo-oxidation and chain scission in a polymer leading to microplastic formation.

Abiotic degradation encompasses chemical-driven processes prevalent in stable, low-energy environments, a context sometimes referred to as static degradation. Key examples include the slow photo-oxidation of plastic films on untilled agricultural soil, the hydrolysis of polyester textiles in saturated sediments, and the thermal ageing of plastics in landfill liners [20]. These processes are often quantified through degradation kinetics; for instance, PE microplastics exposed to TiO_2_ under UV radiation exhibited a degradation rate constant of 2.6 × 10^−7^ *h*^−1^ for nanoparticles [17]. UV exposure frequently initiates photo-oxidation, incorporating oxygen-containing functional groups like ketones and carboxylic acids into the polymer matrix, which further promotes chain cleavage [21]. The process of UV-driven photo-oxidation, which initiates the fragmentation of many common plastics, involves a series of well-defined chemical transformations. The key steps from initial radical formation to the generation of oxygenated chain-end groups that promote brittleness are schematically summarized in Figure 1.

For polymers with hydrolysable bonds, such as PET and PU, hydrolysis, the reaction with water molecules, cleaves the polymer backbone [22]. Concurrently, mechanical abrasion from physical forces like wave action, wind, and sediment friction continually wears down plastic surfaces, while thermal degradation at elevated temperatures can accelerate these chemical breakdown processes [23,24]. The degradation kinetics of plastics vary significantly depending on the polymer type, environmental trigger, and specific conditions. To provide a comparative overview and contextualize the constant rate discussed above, Table 3 summarizes representative quantitative data on degradation and fragmentation for common polymers under key abiotic and biotic triggers, including photocatalytic UV, microbial action, and solar radiation-driven fragmentation.

Biotic degradation involves the action of living organisms, primarily microorganisms. The initial step often involves the colonization of the plastic surface by bacteria, fungi, and algae to form biofilms [25,26]. These microbial communities can then facilitate microbial degradation by producing extracellular enzymes that break down polymer chains into smaller oligomers and monomers, which can be assimilated and mineralized [19]. A highly specific form of this is enzymatic degradation, where enzymes such as hydrolases (e.g., PETase, MHETase for PET), cutinases, esterases, and lipases directly catalyze the hydrolysis of specific bonds in the polymer chain [27,28].

### 2.4. Factors Influencing Degradation Rates

The rate and pathways of plastics degradation to microplastics are not uniform but are modulated by several intrinsic and extrinsic factors. The polymer type and composition are paramount; plastics with carbon-carbon backbones (e.g., PE, PP, PS) are generally more recalcitrant than those with heteroatoms (e.g., PET, PU, PA) that are susceptible to hydrolysis [17,29].

Environmental conditions, such as temperature, UV radiation, and pH, also exert a strong influence on degradation rates and pathways. Higher temperatures accelerate chemical reaction rates and microbial activity [30], while the presence of water facilitates hydrolysis and affects product formation [31]. The intensity of UV radiation directly controls photodegradation kinetics [32], and oxygen availability determines whether degradation proceeds via aerobic or anaerobic pathways [33]. Furthermore, pH levels can modulate enzyme activity and the rate of hydrolytic reactions [34].

The composition and metabolic activity of local microbial communities are crucial for biotic degradation. The rhizosphere microbiome and soil fauna like earthworms and mealworms can significantly enhance bioremediation by ingesting particles and hosting specialized gut microbiota [35]. However, the physical characteristics of the particle itself, namely its surface area and size, are critical. Smaller particles present a larger surface-area-to-volume ratio, increasing their accessibility to UV radiation, water, hydrolytic agents, and microbial colonization, which can paradoxically enhance degradation rates while also increasing their bioavailability and potential ecological impacts [25].

## 3. Microplastics Generation: A Chemical Engineering Perspective

Building upon the detailed classification of primary and secondary sources, a chemical engineering perspective is essential for fundamentally understanding the mechanisms of microplastic generation and for developing innovative, sustainable solutions to mitigate their release. Microplastics, defined as plastic particles smaller than 5 mm, are ubiquitous environmental contaminants whose persistence is a direct consequence of the very material properties, chemical inertness, and durability that make plastics so useful [36,37,38,39]. Chemical engineers, with their expertise in polymer science, material properties, and process engineering, are uniquely positioned to address this challenge by linking molecular-scale interactions to macroscopic industrial processes and environmental fate [38].

### 3.1. Understanding Microplastic Formation

The generation of microplastics is governed by the interplay between polymer material properties and environmental forcing fields, operating through two primary pathways: chemical degradation and mechanical fragmentation [39].

Chemical degradation involves bond-scission reactions that embrittle the plastic matrix. The dominant mechanism for common polyolefins (PE, PP) and polystyrene (PS) is photo-oxidation initiated by ultraviolet (UV) radiation. This process leads to backbone scission and the formation of oxygenated groups (e.g., carbonyls), progressively reducing molecular weight and material integrity [40]. For polymers with hydrolysable backbone bonds, such as polyesters (PET) and polyamides (PA), hydrolysis catalyzed by water, heat, or pH extremes is a critical degradation route.

Mechanical fragmentation results from the physical wear and abrasion of plastic items during use or in environmental weathering. This pathway is a major source of primary MPs (e.g., tire wear, textile fibers) and accelerates the release of secondary MPs from already embrittled plastic debris. Release kinetics depend on applied stress, material toughness, and the presence of fillers. Studies demonstrate that everyday mechanical action on products like bottles, packaging, and textiles can generate significant quantities of micro- and nano-plastics [41,42].

Material properties critically modulate these processes. The polymer’s backbone chemistry determines its susceptibility to specific reactions. Furthermore, the morphology of semi-crystalline polymers creates structural heterogeneities; the less-ordered amorphous regions between crystalline lamellae act as preferential sites for crack initiation and propagation under stress, influencing the final size distribution of generated particles [43,44].

The role of additives and chemical leaching profoundly amplifies the environmental impact of microplastics. Additives such as plasticizers (e.g., phthalates), flame retardants (e.g., brominated compounds), fluorinated substances (PFAS), and stabilizers are not covalently bound to the polymer. Their leaching potential, governed by diffusion coefficients and environmental conditions (temperature, pH), transforms microplastics into mobile point sources of hazardous chemicals. As fragmentation increases surface area, it accelerates the release of these embedded compounds, complicating risk assessment and remediation [14,45].

### 3.2. Chemical Engineering Solutions and Opportunities: Linking Processing to Microplastic Generation

Addressing microplastic generation at source requires chemical engineering strategies that intervene across the material lifecycle, from polymer design to end-of-life processing, thereby linking material properties directly to environmental fate.

Sustainable Material Design aims to reconfigure the inherent conflict between product durability and environmental persistence. This involves (i) designing polymers for controlled degradation, incorporating enzymatically or hydrolytically cleavable bonds that facilitate complete mineralization under specific end-of-life conditions (e.g., industrial composting), rather than uncontrolled environmental fragmentation [46]; and (ii) enhancing product durability to minimize wear, such as developing more abrasion-resistant textile fibers and tire formulations to reduce the generation of primary microplastics during use [47]. A critical parallel effort is the development of benign additives (e.g., non-toxic plasticizers, stabilizers) to reduce the chemical hazard burden if and when degradation occurs [48,49]. These strategies must be applied judiciously; for example, while biodegradable polymers are a target for single-use items, their use in durable goods or in agriculture (e.g., mulching films) requires careful consideration of in-service stability versus post-use degradability.

Advanced Recycling and Waste Processing are essential to capture and repurpose plastics before they can fragment into secondary MPs. This is particularly crucial for managing complex waste streams like mixed plastics, textiles, and microplastic-laden sewage sludge. Mechanical recycling, while valuable, is limited by polymer incompatibility and quality loss. Chemical recycling processes such as pyrolysis (thermal conversion to oils) [50,51], solvolysis (catalytic depolymerization to monomers) [52], and hydrogenolysis (catalytic hydrocracking to lubricants) [53] break plastics down to their molecular constituents, enabling the production of new, high-quality materials and offering a pathway for complex or contaminated streams unsuitable for mechanical recycling. For MPs already present in waste streams (e.g., in wastewater sludge), catalytic conversion methods like photocatalysis can degrade polymer chains [54]. These processing technologies, integrated with robust collection systems, are key to diverting plastics from open environments where they would otherwise weather into secondary microplastics [55].

Effective waste management and a circular economy are foundational to mitigating microplastic pollution [56]. This begins with the establishment of universal and efficient waste collection systems, which is the critical first barrier to prevent plastic littering and open dumping primary pathways for the generation of secondary microplastics. Subsequently, for collected waste streams, improved sorting and separation technologies are required to enable high-quality recycling and advance circularity [57]. Enhancing wastewater treatment is essential, as WWTPs are a significant source of importance; improving removal efficiency through advanced physical (e.g., membrane filtration), chemical (e.g., coagulation), and biological methods is a key engineering challenge [58]. Furthermore, superior landfill management practices are needed to prevent plastic waste from escaping and degrading into microplastics in the environment [59].

Finally, advancing research and characterization is critical. This involves developing advanced analytical techniques (e.g., standardized methods using FTIR, Raman, Py-GC/MS) to reliably identify and quantify nano-plastics in complex matrices [60]. It also requires modeling and prediction using machine learning tools like random forest (RF), gradient boosted decision trees (GBDTs), and XG Boost to forecast generation rates under various scenarios, thereby informing risk assessment and policy [61].

By integrating deep knowledge of polymer science, reaction engineering, and process design, chemical engineers are developing the innovative and sustainable solutions needed to tackle the complex, multi-scale challenge of microplastic pollution [61].

## 4. Transport, Fate, and Interactions with Pollutants

**Figure 2:**
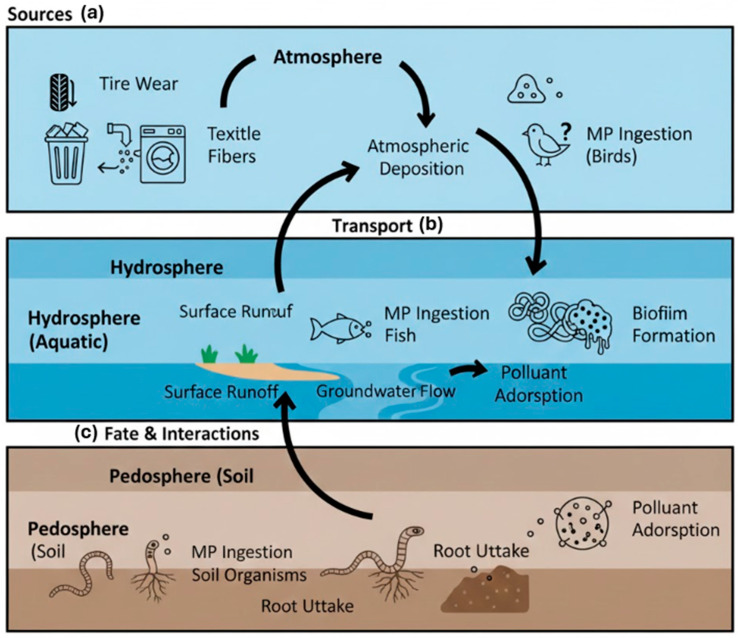
Conceptual schematic of microplastic environmental fate and transport. The figure illustrates key pathways across major environmental compartments: (**a**) Primary emission sources (e.g., tire wear, textile fibers, mismanaged waste) and Inter-compartmental transport vectors (e.g., atmospheric deposition, surface runoff, groundwater flow); (**b**) Key interaction processes within compartments, including ingestion by biota (fish, birds, soil organisms) and Surface modification processes, such as biofilm formation and aggregation; and (**c**) Chemical interactions, notably the adsorption of co-pollutants onto MP surfaces.

The environmental impact of microplastics (MPs) and nano-plastics (NPs) is governed not only by their sources and formation but also by their subsequent transport, fate, and ability to interact with other pollutants. The environmental journey of micro and nano-plastics is a complex, multi compartmental process that determines their ultimate distribution, persistence, and ecological impact. Their transport involves physical movement across and between atmospheric, aquatic, and terrestrial systems, while their fate refers to their long-term destination and transformation within these compartments. A critical aspect of their impact is their role as vectors, interacting with and concentrating co-occurring pollutants. To visualize this integrated lifecycle, Figure 2 presents a schematic overview of the primary sources, key transport pathways, and major interaction mechanisms of MPs/NPs in the environment. The following subsections detail the physicochemical properties governing these processes.

### 4.1. Physicochemical Properties Governing Environmental Fate

The initial distribution is dynamically altered by biofouling the attachment of microorganisms and organic matter, which increases the effective density of buoyant particles, causing them to sink over time [62,63].

Particle size and shape are equally critical properties dictating mobility, bioavailability, and ecological impact. Size directly influences ingestion by organisms; larger MPs can cause physical blockages in digestive tracts, while smaller NPs (<100 nm) can cross biological barriers, including cell membranes and the placenta, posing risks for systemic toxicity [64,65,66,67,68]. Smaller particles also exhibit higher mobility, traveling greater distances through water, soil, and air [69]. Shape further modulates these interactions. Fibers, prevalent from textiles and fishing gear, are easily transported by wind and runoff [62,70]. Fragments from the breakdown of larger items are common in marine environments, while films and pellets are significant in soils from agricultural and industrial sources [62]. The shape also influences the physical harm caused upon ingestion, with sharp, irregular, or fibrous shapes causing more damage than spherical ones [71].

Surface properties are paramount for colloidal stability and pollutant interactions. The surface charge, quantified by the zeta potential (ζ), controls particle aggregation and dispersion, influencing their mobility and interaction with biological membranes [72]. Wettability, or hydrophobicity, determines the particle’s affinity for water. Most virgin plastics e.g., polyethylene (PE), polypropylene (PP), and polystyrene (PS) are highly hydrophobic, a property that can facilitate the adsorption and partitioning of hydrophobic organic contaminants (HOCs) like polycyclic aromatic hydrocarbons (PAHs) and polychlorinated biphenyls (PCBs), though the sorption capacity is highly variable and depends on polymer type, contaminant properties (e.g.,the octanol-water partition coefficient log KOW), and environmental conditions [73,74]. It is important to note that some high-concentration data cited in the literature derive from studies using plastic pellets as deliberate passive samplers, which may not represent typical environmental MPs. Furthermore, while MPs can act as contaminant vectors, their total sorption capacity in a given environmental compartment is often outweighed by that of natural organic matter in soils and sediments. Environmental weathering through UV radiation, oxidation, and mechanical abrasion alters these surface properties by introducing oxygen-containing functional groups (e.g., carbonyl, hydroxyl), increasing hydrophilicity and changing the surface charge [75]. This aged, oxidized surface provides an ideal substrate for microbial colonization and biofilm formation (the “plastisphere”) [76] and enhances the adsorption capacity for metal ions by creating new binding sites [77]. These evolving surface functional groups also mediate biological interactions by affecting protein corona formation, which in turn dictates cellular uptake and potential toxicity [78].

### 4.2. Microplastics and Nano-Plastics as Contaminant Vectors

The role of MPs/NPs as vectors for other persistent pollutants, such as heavy metals (Cd, Pb, Cu, Zn, Cr, Hg) and hydrophobic organic contaminants (HOCs like PAHs, PCBs, and PAEs), significantly amplifies their environmental threat [79,80,81]. The sorption mechanisms facilitating this are multifaceted. Hydrophobic partitioning is the primary driver for HOCs, where non-polar compounds diffuse into the non-polar polymer matrix [80]. Surface sorption encompasses various interactions, including hydrogen bonding, π–π interactions, electrostatic forces, and van der Waals forces [82]. Additionally, pore filling within the porous structure of some plastics can trap contaminant molecules [82].

The extent of sorption is governed by a three-way interaction between the properties of the plastic, the contaminant, and the environment. Key factors influencing sorption include:

MP/NP Properties: Smaller particle size provides a larger specific surface area for sorption, though aggregation can reduce this effect [83,84]. Polymer type dictates inherent polarity and crystallinity; amorphous regions above the glass transition temperature (e.g., in PE, PP) allow greater contaminant diffusion than glassy polymers (e.g., PVC) [85,86,87]. Weathering increases surface area, roughness, and oxygen content, typically enhancing sorption for metals and hydrophilic organics but potentially reducing it for HOCs due to increased polarity [86].

Contaminant Properties: The hydrophobicity commonly measured by the octanol-water partition coefficient (log KOW) of organic compounds and the charge/speciation of metal ions are primary determinants of their affinity for plastic surfaces [88,89]. Specific functional groups on contaminants can enable hydrogen bonding or π–π interactions [89].

Environmental Conditions: Solution chemistry exerts a strong influence. pH affects the ionization state of both contaminants and the plastic surface, altering electrostatic interactions [90]. High ionic strength can lead to competitive inhibition for sorption sites [91]. Dissolved organic matter (DOM) can compete for sites or act as a bridge to facilitate binding [92], while the presence of multiple contaminants can lead to competitive adsorption effects [93]. These governing factors are illustrated by specific experimental findings. For example, Guo et al. (2020) demonstrated that the sorption capacity of cadmium (Cd) onto polyethylene microplastics increased significantly after UV aging, due to the formation of oxygen-containing functional groups that provided new binding sites [85,86]. Similarly, the sorption of phenanthrene (a PAH) onto polystyrene was shown to be highly dependent on particle size, with smaller microplastics exhibiting greater adsorption capacity per unit mass [84]. Furthermore, competitive sorption studies in marine environments have shown that polyethylene can simultaneously accumulate multiple persistent organic pollutants like PCBs and DDT, with their relative affinities governed by hydrophobicity (log KOW) [93].

### 4.3. Modeling Transport from a Process-Engineering Lens

Predicting the complex transport and fate of MPs requires advanced computational tools. Computational Fluid Dynamics (CFD) has emerged as a powerful methodology for this purpose, analyzing fluid behavior by solving the governing equations for the conservation of mass, momentum, and energy [94,95]. CFD simulations minimize the need for extensive experimental trials by providing high-resolution insights into MP trajectories, dispersion, and accumulation hotspots in aquatic environments [95].

Within CFD frameworks, several numerical models are integrated to simulate MP transport accurately. The Volume of Fluid (VOF) model is often used to track free-surface flows, crucial for modeling surface water bodies. The Discrete Phase Model (DPM) treats MPs as a discrete phase of particles, calculating their trajectories through the simulated fluid flow field, accounting for forces like drag and buoyancy. To capture the turbulent nature of most environmental flows, turbulence models like the k-omega SST are employed to provide a realistic representation of the mixing and shear forces that disperse and transport particles [94]. This process-engineering approach is essential for scaling laboratory findings to real-world ecosystems and for designing effective intervention and remediation strategies.

## 5. Detection and Characterization of Microplastics: Analytical Engineering Approaches

Accurately quantifying and characterizing microplastics (MPs) and nano-plastics (NPs) is a fundamental prerequisite for assessing environmental contamination levels, evaluating the efficacy of treatment processes, and tracing pollution sources. This requires a sophisticated analytical engineering approach to a systematic methodology for selecting, integrating, and validating techniques based on the specific challenges of the sample and the required data output.

### 5.1. The Analytical Engineering Challenge: From Field Sampling to Data Interpretation

The accurate detection and characterization of microplastics (MPs) and nano-plastics (NPs) present a formidable analytical engineering challenge, extending far beyond the capabilities of any single laboratory instrument. This challenge is defined by a multi-step workflow where each stage introduces potential bias and uncertainty, demanding a systematic, fit-for-purpose approach.

Sampling and Pre-treatment: The first and often most critical step is obtaining a representative sample from a heterogeneous environmental matrix (water, soil, air, biota). Sampling design must account for particle distribution, which is influenced by density, currents, wind, and aggregation. Subsequent sample processing including digestion of organic matter, density separation, and filtration aims to isolate plastic particles while minimizing loss or contamination. These pre-treatment protocols are not universal; they must be tailored to the sample matrix and target particle size, representing a significant source of methodological variability that can compromise data comparability across studies.

The Selection of an Analytical Technique is not a mere choice of the most advanced tool but an engineering trade-off based on the analysis objective (e.g., particle count, polymer identification, mass concentration), the sample characteristics (matrix complexity, expected concentration, size range), and practical constraints (cost, throughput, expertise). No single technique provides a complete picture; a multi-method approach is typically required. For instance, optical methods can enumerate particles but cannot identify polymer type, while spectroscopic methods identify polymer but may miss particles outside their size detection range.

Therefore, the ‘analytical engineering approach’ advocated here involves designing an integrated workflow that consciously addresses these sampling and selection challenges to generate reliable, interpretable data. The following subsections detail the common and emerging techniques available, with their suitability for different stages of this workflow summarized in Table 3.

### 5.2. Common Analytical Techniques

#### 5.2.1. Physical Characterization and Sizing

The initial step in MP analysis involves physical characterization, determining size, shape, and surface morphology, which provides clues about origin and environmental history. Visual and optical microscopy (stereo, fluorescence) offer a low-cost, rapid screening method for particles > 100 µm but are prone to misidentification of non-plastic particles [96]. Staining with hydrophobic dyes (e.g., Nile Red) enhances contrast and specificity for fluorescence-based detection and counting. For higher-resolution topological imaging, electron microscopy (SEM, TEM) is invaluable. However, SEM alone cannot chemically identify polymers; it only reveals morphology. Therefore, SEM is typically coupled with Energy-Dispersive X-ray Spectroscopy (EDS) for elemental analysis, which helps distinguish carbon-based plastics from inorganic particles but cannot differentiate polymer types. This combination is crucial for “clear distinction” from some, but not all, particulate impurities [96].

For robust size distribution analysis, laser diffraction provides fast, high-resolution data for particles from 0.04 µm to 2 mm. At the nanoscale, Dynamic Light Scattering (DLS) estimates the hydrodynamic diameter of NPs in suspension, though its accuracy is compromised by sample polydispersity and the presence of non-plastic colloids [96].

#### 5.2.2. Chemical Composition Identification

Determining polymer type is essential for source apportionment and fate studies. Vibrational spectroscopy techniques are the workhorses for chemical identification. Micro-Fourier Transform Infrared (µ-FTIR) spectroscopy identifies polymers by their characteristic IR absorption bands, with extensive spectral libraries available for matching. Its limitations include water interference and a practical size detection limit of ~10–20 µm. Micro-Raman (µ-Raman) spectroscopy, based on inelastic light scattering, complements FTIR with higher spatial resolution (<1 µm) and better suitability for aqueous samples, but it can suffer from fluorescence interference that masks the polymer signal [96].

Thermal analysis techniques, such as Thermogravimetric Analysis (TGA) and Differential Scanning Calorimetry (DSC), study mass loss and thermal transitions, providing information on polymer composition and thermal stability. For definitive molecular identification and additive analysis, mass spectrometry (MS) is employed, but not via standard GC-MS or LC-MS of intact particles. The primary MS method for polymers is Pyrolysis-Gas Chromatography-Mass Spectrometry (Py-GC-MS), which thermally decomposes the plastic into volatile fragments that are then separated and identified, providing a unique fingerprint for polymer type and often for organic additives [96].

#### 5.2.3. Quantitative Analysis

Accurate quantification is critical for risk assessment and remains a major challenge. Quantification can be based on particle number or polymer mass, requiring different approaches. (i) Particle Number: Automated µ-FTIR or µ-Raman imaging systems can identify and count thousands of particles in a sample filter scan, providing size-resolved particle number concentrations. This approach is powerful but time-consuming and has size detection limits. (ii) Polymer Mass: Thermogravimetric Analysis (TGA) measures the mass loss associated with polymer thermal decomposition, providing a bulk mass concentration that is independent of particle shape or size. Py-GC-MS can also be quantitative when combined with appropriate calibration, reporting mass concentrations of specific polymers. But standard GC-MS and LC-MS are not used to quantify intact MP particles. They are, however, crucial for quantifying leached additives (e.g., plasticizers, flame retardants) or specific monomer markers after chemical digestion. (iii) Nano-plastic and Elemental Analysis: Single-Particle Inductively Coupled Plasma Mass Spectrometry (SP-ICP-MS) is a powerful technique mentioned in the literature, but its primary application is for metal-containing nanoparticles or for tracing metals adsorbed onto plastics. It is not a direct method for quantifying carbon-based polymer mass, though it can be used with metal tags or for plastics containing inorganic fillers.

Non-specific index methods like Total Organic Carbon (TOC) can indicate overall organic load, including plastics, but lack any specificity for MPs.

The selection of a quantitative strategy is a critical engineering decision based on the required information (number vs. mass), the expected size range, the complexity of the matrix, and available resources. The advantages and limitations of key techniques are synthesized in Table 4 to guide this selection.

### 5.3. Emerging Technologies and Future Directions

The field of microplastic analysis is rapidly advancing to overcome persistent challenges in sensitivity, selectivity, throughput, and field applicability. Beyond the refinement of existing techniques, future progress hinges on the convergence of spectroscopy, materials science, and data analytics.

Integration of Artificial Intelligence and Machine Learning (AI/ML): A transformative trend is the application of AI/ML to automate and enhance data analysis. Machine learning algorithms are being developed to rapidly classify particle spectra from µ-FTIR and µ-Raman imaging, significantly reducing analysis time and minimizing human bias [98]. Furthermore, AI is being leveraged to manage complex datasets, model MP fate and transport, and optimize mitigation strategies, representing a paradigm shift toward data-driven environmental management [99].

Advanced Nanoscale and Single-Particle Characterization: For nanoplastics, the push is toward correlative microscopy that combines high-resolution topological data with definitive chemical identification. Techniques like AFM-IR spectroscopy and the continued development of high-resolution mass spectrometry methods (e.g., ASAP-MS) are crucial for understanding the composition, surface properties, and potential toxicity of particles at the sub-micron scale [97].

Field-Deployable and High-Throughput Screening: There is a growing demand for technologies that move analysis from the lab to the field. Portable NIR/VIS-NIR spectrometers and the development of rapid chemical sensor arrays aim to provide real-time, on-site screening for MP pollution hotspots. The ultimate goal is the creation of standardized, automated platforms capable of processing large environmental sample sets with minimal pre-treatment.

The critical research needs for the coming years include: (i) establishing standardized protocols for nanoplastic extraction and analysis; (ii) developing certified reference materials for method validation; (iii) bridging the gap between laboratory detection and in situ monitoring; and (iv) fostering interdisciplinary collaboration to translate fundamental advances into robust, engineered solutions for environmental monitoring.

## 6. Engineered Treatment and Removal Technologies: Unit Operations and Process Integration

**Figure 3:**
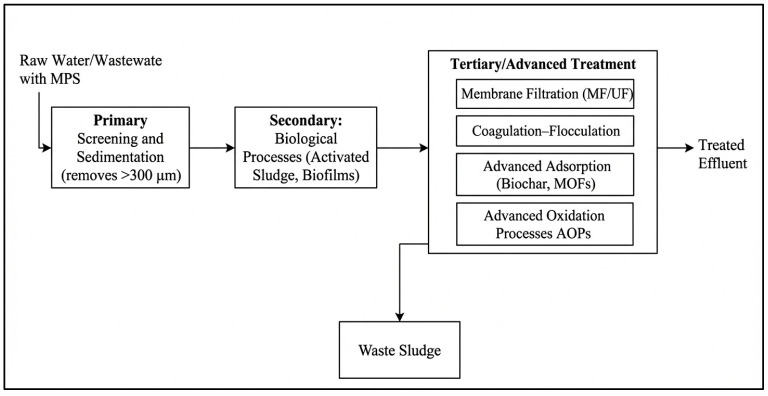
Microplastic Removal: Unit Operations & Treatment Trains.

The effective management of microplastic (MP) pollution necessitates robust engineering solutions within wastewater treatment plants (WWTPs), which act as critical barriers between anthropogenic sources and natural ecosystems. The removal of MPs is not the primary design function of conventional WWTPs but occurs because of unit operations targeting other pollutants. The efficacy of these processes depends on the integration of physical, biological, and chemical mechanisms, each presenting distinct engineering trade-offs between removal efficiency, energy consumption, and cost. The removal of microplastics from engineered water systems can be systematically analyzed through the chemical engineering concept of unit operations discrete steps based on physical separation principles. Key operations include preliminary screening (size exclusion), primary sedimentation (gravity settling), dissolved air flotation (buoyancy), and secondary/tertiary filtration (size exclusion and adsorption). These are primarily mass transfer and separation operations. To provide a clear overview of this integrated process, Figure 3 presents a generalized treatment train, categorizing technologies from preliminary screening to advanced tertiary processes. This framework allows for the evaluation of technological trade-offs in terms of removal efficiency, energy demand, and waste generation. The following subsections detail the specific mechanisms within conventional wastewater treatment plants and discuss the critical issue of the final fate of removed microplastics, emphasizing the need for holistic process integration.

### 6.1. Microplastic Removal Mechanisms in WWTPs

WWTPs employ a multi-stage treatment train, with each stage contributing to MP removal through specific unit operations. Preliminary treatment serves as the first line of defense, utilizing physical processes like screening and grit removal. Bar screens capture large debris, including MPs larger than the mesh size (typically > 6 mm), while grit chambers are designed to settle out dense inorganic materials but can also remove some denser plastic fragments incidentally [100,101]. The efficacy of microplastic removal varies significantly across different unit operations within WWTP, as each stage targets particles based on size, density, and surface properties. The typical performance ranges and primary mechanisms for key treatment processes are quantitatively summarized in Table 5.

Primary treatment follows, relying on physical separation through sedimentation in primary clarifiers. This process is highly effective for removing larger and denser MPs via gravitational settling, while buoyant particles are removed through skimming. The efficiency of this stage is governed by the particle’s density, size, and shape, making it a crucial step for reducing the overall MP load before biological treatment [100].

Secondary treatment introduces biological processes, primarily the activated sludge process, where microorganisms metabolize organic matter. MPs are removed here through two key mechanisms: bio-flocculation, where particles become entrapped within the dense microbial flocs, and direct ingestion or enzymatic degradation by specific microorganisms. Research has identified various bacterial and fungal species capable of degrading polymers; for example, *Ideonella sakaiensis* produces enzymes (PETase) that hydrolyze PET, while certain strains of *Pseudomonas* and *Bacillus* can degrade polyethylene and polypropylene. However, the application of these specific degrader organisms in full-scale municipal wastewater treatment remains limited due to challenges in maintaining optimal consortia and process control. Most MP removal in conventionally activated sludge is attributed to non-specific bio-flocculation. The resulting biomass is then separated from the treated water in secondary clarifiers, effectively sequestering the incorporated MPs. The removal efficiency in this stage is influenced by the sludge retention time (SRT) and the properties of the MPs themselves [100]. To achieve the highest effluent quality, tertiary (or advanced) treatment processes are employed. These technologies are particularly effective for capturing the smaller MPs that bypass earlier stages; (i) Membrane Bioreactors (MBRs), which integrate biological treatment with low-pressure membrane filtration (e.g., microfiltration with pore sizes of 0.1–0.4 µm), represent one of the most effective technologies, demonstrating exceptional removal efficiencies of 99.4% to 99.9% [100], (ii) Granular Media Filtration (e.g., rapid sand filters) can reduce MP concentrations in secondary effluent by over 50%, acting as a depth filter to capture particles [102], (iii) Coagulation/Flocculation involves adding chemical coagulants (e.g., alum, ferric chloride) to destabilize colloidal particles, including MPs, and aggregate them into larger, settleable flocs. The related process of Electrocoagulation, which uses sacrificial anodes to generate coagulants in situ, offers a chemical-free alternative with similar aggregation goals [102], (iiii) Advanced Oxidation Processes (AOPs), such as ozonation and UV/H_2_O_2_, function differently by not removing but rather degrading MPs through oxidative chain scission, breaking them down into smaller, and ideally more biodegradable, molecules [103].

### 6.2. Fate of Removed Microplastics and Process Integration

Critical engineering and environmental considerations are the fate of MPs removed from the wastewater stream. The vast majority are transferred to and concentrated in the sewage sludge. Studies report substantial MP concentrations in sludge, ranging from 720 to 14,900 particles per kg (wet weight) and 1000 to 170,900 particles per kg (dry weight), with synthetic fibers constituting 63–90% of this load [100]. This creates a significant waste management challenge. The common practice of applying treated sludge (biosolids) as agricultural fertilizer reintroduces these concentrated MPs into terrestrial ecosystems, creating a potential pathway for soil accumulation and subsequent transport to aquatic systems via runoff or wind erosion [100].

Therefore, process integration must extend beyond mere removal efficiency within the water line. A holistic chemical engineering approach requires evaluating the entire system: optimizing unit operations in the water treatment train for maximum MP capture while simultaneously developing and integrating solutions for managing the contaminated solid waste stream, thereby closing the loop and preventing secondary environmental release. This imperative for integrated waste management extends beyond the wastewater treatment train. The remediation strategies for terrestrial and atmospheric systems discussed in subsequent sections similarly concentrate MPs into secondary waste streams such as contaminated soil washing effluent, spent bioremediation biomass, or captured filter media from air scrubbers. A holistic chemical engineering approach therefore requires that the development of any MP removal technology must be coupled with a viable pathway for the final containment, destruction, or valorization of the concentrated residual. This system-level perspective is essential for designing truly sustainable and circular mitigation solutions across all environmental compartments.

### 6.3. Mitigation in Terrestrial Systems

Microplastic (MP) accumulation in agricultural and urban soils presents a distinct remediation challenge compared to aquatic systems, requiring strategies tailored to a solid, heterogeneous matrix. Effective mitigation focuses on two primaries, often complementary, approaches: biologically driven degradation and physical separation.

Biologically Driven Degradation: This approach leverages natural metabolic processes to fragment and mineralize plastic polymers. Microbial remediation is the most studied pathway, where bacteria and fungi secrete extracellular enzymes (e.g., hydrolases, oxidases) that depolymerize plastics like polyethylene (PE) and polypropylene (PP) [104]. Efficacy is highly dependent on polymer type, soil conditions (e.g., moisture, pH, oxygen), and the native microbial community, which can be both inhibited and structurally altered by MP presence [105,106]. Strategies to enhance this process include bioaugmentation (adding specific degrader consortia) and bio-stimulation (amending soil with nutrients or co-substrates), which are core concepts in environmental biotechnology [105]. Phytoremediation offers indirect support; plants stabilize soil to prevent MP run off and their root exudates can stimulate microbial activity in the rhizosphere, potentially accelerating biodegradation [107].

Physical Separation and Management: For heavily contaminated sites, engineered physico-chemical methods may be necessary. Soil washing, a unit operation adapted from mining and sediment remediation, uses aqueous solutions (sometimes with surfactants) to separate MPs from soil particles based on density and size. While effective for concentrated point-source contamination, challenges include high water/energy use, chemical addition, and the need to treat the resulting MP-laden wastewater. An integrated chemical engineering perspective therefore evaluates these biological and physical unit operations not in isolation, but within a system that also manages the fate of removed contaminants and optimizes resource use.

### 6.4. Mitigation in Atmospheric Systems

The mitigation of airborne microplastics (AMPs) represents an emerging frontier in pollution control, as research on their sources, behavior, and engineered capture is less developed than for aquatic systems. AMPs originate from sources such as textile wear, tire abrasion, and the resuspension of settled dust, with significant concentrations documented in both indoor and outdoor environments [108,109]. Their small size and low density make them susceptible to long-range atmospheric transport. Effective control strategies must therefore target both primary emissions and ambient concentrations.

From a chemical engineering perspective, mitigation can be conceptualized across two scales: source control and end-of-pipe treatment. At the source, process modifications in industries generating plastic dust or fibers (e.g., textile manufacturing, plastic recycling facilities) can minimize release. This includes enclosure of operations, improved ventilation design, and the use of wet scrubbers to capture particles at the point of generation.

For the capture of AMPs from ambient or indoor air, established particulate matter (PM) control technologies are directly applicable but require validation for plastic particles. Key unit operations include: (i) Filtration: High-Efficiency Particulate Air (HEPA) filters and baghouse filters, which physically capture particles on fabric media, are expected to be highly effective for AMPs given their similarity to other fine aerosols. (ii) Electrostatic Precipitation (ESP): This technology charges particles and collects them on oppositely charged plates, offering a low-pressure-drop solution suitable for high-volume air streams, such as in building HVAC systems or industrial stacks. (iii) Cyclonic Separation: While less effective for sub-micron particles, cyclones could serve as a pre-treatment step to remove larger AMP fragments.

A significant research gap exists in quantifying the removal efficiency of these standard air pollution control devices specifically for AMPs, as their surface properties (e.g., static charge, hydrophobicity) may differ from those of typical mineral dust [108,109]. Furthermore, the fate of AMPs captured in filters or sludge from scrubbers presents a waste management challenge analogous to that of MPs removed from wastewater, necessitating integrated system design. Future work must focus on characterizing AMP emissions, testing the performance of adapted control technologies, and developing monitoring protocols to assess mitigation effectiveness.

## 7. Material Redesign: Biodegradable Polymers and Circular Engineering

The escalating crisis of plastic pollution necessitates a paradigm shift from end-of-pipe treatment solutions to upstream material redesign. This requires, as demonstrated in the study by [110], a fundamental reimagining of polymer lifecycles through the dual lenses of advanced biodegradable materials and circular engineering principles, moving from a linear “take-make-dispose” economy to a regenerative circular model.

### 7.1. Biodegradable Polymers: Promise and Cautions

Biodegradable polymers represent a promising class of materials engineered to decompose through microbial action into benign end products such as water, carbon dioxide, methane, and biomass, offering a stark contrast to the centuries-long persistence of conventional plastics [111,112]. The primary driver for their development is the urgent need to address plastic waste accumulation in natural ecosystems and reduce reliance on finite fossil resources [111]. Among the most promising candidates are polyhydroxyalkanoates (PHAs), a family of polyesters that can be naturally synthesized by microorganisms or derived synthetically from bio-renewable feedstocks [113]. However, the widespread adoption of biodegradable plastics is not without caveats. Their performance properties, such as brittleness and melt-processability, have historically lagged those of conventional polymers, limiting their application. Furthermore, the term “biodegradable” is often misconstrued; efficient degradation typically requires specific conditions found in industrial composting facilities and may not occur at meaningful rates in ambient marine or terrestrial environments, leading to potential accumulation if not managed correctly.

### 7.2. The Role of Chemical Recycling in a Circular Economy

To truly close the loop on plastic materials, end-of-life management must be tailored to the product’s application and likelihood of entering the environment. Chemical recycling emerges as a cornerstone technology for a circular economy, capable of depolymerizing plastic waste back into its constituent monomers [114]. Unlike mechanical recycling, which often leads to downcycling, chemical recycling can produce virgin-quality monomers, enabling closed-loop recycling and decoupling plastic production from fossil feedstocks [115].

Application-Specific Material Strategies: The choice between conventional, recyclable polymers and biodegradable alternatives must be strategic. For durable goods with long service lives and high collection rates (e.g., automotive parts, construction materials), the circular economy priority is to design for durability, repairability, and efficient recycling making chemically recyclable versions of conventional polymers like polyolefins or PET the most viable path. Conversely, for short-lived, high-risk applications where products are difficult to collect or highly likely to be littered (e.g., single-use packaging, agricultural mulching films), biodegradable polymers like polyhydroxyalkanoates (PHAs) offer a critical safety net. For these materials, integrating chemical recycling creates a synergistic system: they can be recovered and chemically recycled into high-value monomers, with their inherent biodegradability serving as a benign final endpoint for any material that escapes the managed waste stream [116]. This application-specific framework ensures that material design aligns with realistic waste management pathways and environmental risk.

A general approach to mitigating microplastic pollution requires evaluating a suite of strategies, from advanced engineering treatments to fundamental material redesign. Each solution presents a unique balance of effectiveness, scalability, and economic viability. A comparative analysis of these leading mitigation strategies is provided in Table 6.

### 7.3. Broader Implications for Bioplastics and Sustainability

The advancement of recyclable and biodegradable polymers carries profound implications for the bioplastics industry and global sustainability efforts that extend far beyond waste management. Enhancing market competitiveness represents a critical factor for adoption. By addressing traditional limitations of bioplastics through advanced synthesis and compounding techniques, these new materials become functionally competitive with conventional plastics [118]. When coupled with the long-term economic advantage of closed-loop chemical recycling, this creates a compelling proposition for industry adoption. Environmental benefits encompass a systemic reduction in ecological impact. The dual strategy of inherent biodegradability and efficient chemical recyclability offers a multi-layered solution to plastic pollution that reduces the carbon footprint and greenhouse gas emissions associated with conventional plastic production [119]. This technological advancement serves as a significant accelerator for the transition to a circular economy. The development of these dual-function materials demonstrates a viable pathway for sustainable resource management where materials are continuously circulated at their highest value, thereby contributing to climate change mitigation and reduced environmental degradation [110,120]. Furthermore, these innovations catalyze broader developments in sustainable polymer design, fostering new materials engineered with not only performance but also end-of-life considerations from the initial design phase [121].

### 7.4. Trends in Sustainable Additives

The transition to a circular and less hazardous plastics economy necessitates innovation not only in polymer backbones but also in the chemical additives that impart functionality (e.g., flexibility, flame resistance, color, stability). Regulatory pressures and consumer demand are driving a significant shift away from legacy additives of concern, such as certain phthalate plasticizers and halogenated flame retardants, due to their persistence, toxicity, and potential to leach into the environment throughout the plastic lifecycle [14,16,45].

This shift is fostering the development and adoption of sustainable additive alternatives. These include (i) Bio-based and non-toxic plasticizers derived from sources like citric acid or vegetable oils, designed to minimize endocrine-disrupting effects and improve compatibility with biodegradation processes. (ii) Inherently safer flame retardants, including mineral-based (e.g., aluminum tri-hydroxide) and certain phosphorus/nitrogen-based compounds, which aim to reduce bioaccumulation potential. (iii) Natural stabilizers and colorants that reduce the load of heavy metals and synthetic organic compounds.

The strategic redesign of additives is critical, as their release can be a significant environmental burden [14,48]. For biodegradable polymers, additives must be selected to not inhibit microbial activity during composting. For chemical recycling, additives should be designed to either decompose cleanly during depolymerization or be easily separable to avoid contaminating the monomer stream. Therefore, a holistic approach to sustainable plastic design must integrate the engineering of the polymer, its additive package, and its intended end-of-life pathway from the outset [45,48].

## 8. Modeling, Techno-Economic Analysis, and Life-Cycle Assessment

A comprehensive assessment of microplastic mitigation strategies requires the integration of sophisticated modeling approaches, rigorous economic analysis, and holistic environmental evaluation. This multi-disciplinary framework is essential for guiding policy decisions, optimizing engineering solutions, and ensuring sustainable outcomes across the entire plastic lifecycle.

### 8.1. Kinetic and Transport Modeling

Predicting microplastic behavior in complex environmental systems necessitates advanced modeling approaches that capture their formation, transport, fragmentation, and degradation kinetics [122,123]. Fate modeling provides a quantitative framework for predicting the distribution and persistence of plastics in the environment [124]. These models incorporate several critical mechanisms: redistribution, which tracks the movement of microplastics within and between environmental compartments, such as the transport from terrestrial systems to aquatic ecosystems via surface runoff and erosion processes; fragmentation, representing the physical breakdown of larger plastic items into smaller microplastics without chemical mineralization; and degradation, which describes the chemical breakdown of polymer chains leading to reduced molecular mass and eventual mineralization [122].

Complementing fate models, exposure and effect modeling establishes the connection between microplastic emissions and their environmental impacts, particularly within life-cycle assessment (LCA) frameworks. This approach relies on characterization factors (CFs) that integrate a fate factor (FF) to predict environmental distribution, an exposure factor (XF) to estimate organism contact, and an effect factor (EF) to quantify ecological damage per unit of exposure [122,124].

### 8.2. Techno-Economic Analysis (TEA)

Techno-economic analysis provides a critical evaluation of the economic viability and cost-effectiveness of technologies and strategies for microplastic management. This comprehensive assessment encompasses costs associated with plastic production, waste collection, recycling infrastructure, and the development of alternative materials [125]. To move beyond conceptual frameworks, illustrative cost data is essential for understanding the economic landscape and trade-offs between conventional and emerging pathways.

#### 8.2.1. Polymer Production and End-of-Life Management Costs

Production costs for both conventional (e.g., polypropylene, PP) and bio-based (e.g., polylactic acid, PLA) polymers vary significantly with factors such as part weight, production lot size, and raw material prices. Notably, when lot size and part weight are equal, PLA can outperform PP in both economic and environmental terms under optimized waste management scenarios. For example, a cradle-to-grave analysis comparing PP and PLA demonstrated that, at a province scale, shifting from a baseline of 90% landfill/open incineration for PP to a system emphasizing recycling and composting for PLA resulted in a 63% economic gain and a 39% reduction in global warming potential (GWP). At the city scale, replacing landfilled PP with a mix of recycling and incineration with energy recovery (IwE) for both polymers led to a 22% economic gain and a 26% reduction in GWP. However, these improved waste management activities (e.g., sorting, processing) can also increase emissions of certain pollutants, such as high carcinogens (+137%) and total air toxics (+9%) [126].

Order-of-magnitude end-of-life (EOL) management costs per kilogram of polymer, derived from scenario-based LCA/TEA studies, highlight the economic variability and critical role of system design [126]: (i) Open Incineration (OI): $0.01–$0.25 for PP, $0.002–$0.08 for PLA (town to province scale). (ii) Incineration with Energy Recovery (IwE): $0.29–$0.44 for PP, $0.29–$0.31 for PLA (city to province scale). (iii) Landfilling and Mechanical Recycling: While specific unitary costs for these pathways were not isolated in the primary source, the integrated scenario analyses confirm that landfilling is typically the lowest-cost disposal option, whereas mechanical recycling introduces processing costs that are offset by the value of the recycled material and avoided virgin production [126]. External cost internalization is a decisive factor: For instance, healthcare sector costs attributed to microplastics acting as toxin vectors are estimated at $16.5 per kg of microplastics at the province scale, illustrating the significant hidden liabilities of pollution [126].

#### 8.2.2. Recycling Technologies and Economic Viability

Traditional mechanical recycling remains the most cost-effective and widely adopted method for mono-material streams, but its efficiency and economics are constrained by material degradation and contamination levels. Advanced recycling technologies, such as chemical (e.g., depolymerization) and biological recycling, can process mixed and contaminated plastics, converting them back into monomers for new polymer production. While these methods currently face higher capital and operational costs, ongoing innovation and economies of scale are expected to improve their economic competitiveness. Mechanical innovations, including AI-based sorting and compatibilization strategies, are crucial for enhancing the quality and market value of recycled materials, directly improving the economic calculus of recycling operations and supporting the transition to a circular economy [125].

#### 8.2.3. Key Economic Trends and Implications

Synthesizing the TEA literature reveals several critical trends:

(i) Scale and System Design are Paramount: Economies of scale and a strategic shift from linear disposal (landfilling, open burning) to managed EOL pathways (recycling, composting, IwE) are essential for achieving cost-competitive and sustainable polymer use.
(ii) Full-Cost Accounting is Necessary: Significant externalities, such as healthcare costs from microplastic pollution ($16.5/kg MP), must be internalized in TEAs to reflect the true societal cost of plastic products and justify investments in mitigation.
(iii) Innovation Requires Support: Technological advancements in both mechanical and advanced recycling are critical for reducing costs and environmental impacts but require consistent policy support (e.g., extended producer responsibility, recycled content mandates) and multi-stakeholder collaboration for widespread adoption [125,126].

It is critical to note that the cost figures presented are highly variable and context-dependent. They fluctuate based on geographic location, scale of operation, local policy incentives, and the quality of the waste feedstock. The values serve as illustrative, order-of-magnitude estimates to highlight the economic dimensions, trade-offs, and the importance of system-level analysis in microplastic management strategies.

### 8.3. Life-Cycle Assessment (LCA)

**Figure 4:**
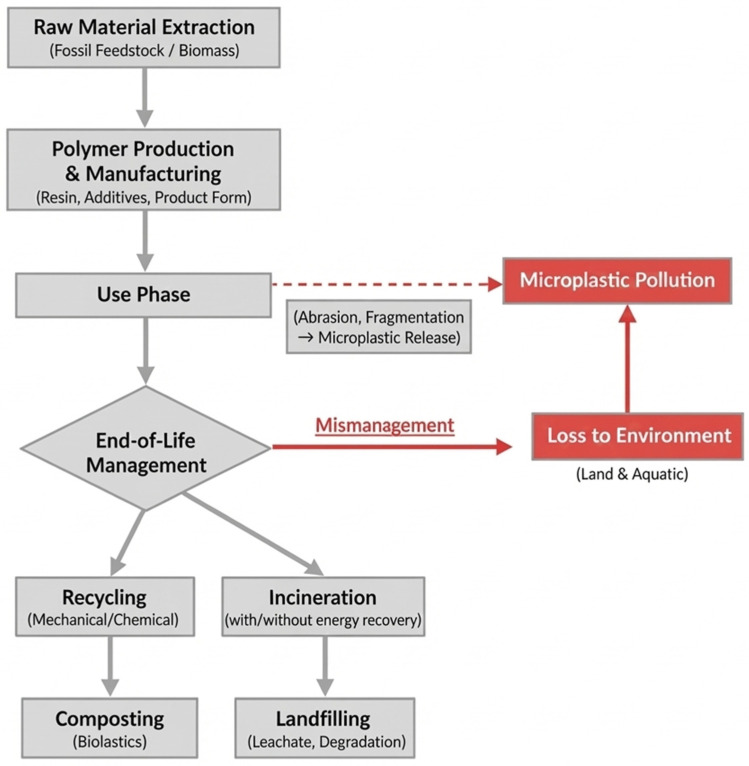
Generalized lifecycle stages of conventional and bio-based plastics, highlighting pathways to microplastic pollution.

Life-cycle assessment serves as a systematic methodology for evaluating the environmental impacts of products and processes across their entire lifecycle. For plastics, this canonical lifecycle encompasses four primary stages: (i) Raw Material Extraction & Feedstock Production (e.g., petroleum refining or cultivation of biomass); (ii) Polymer Production & Manufacturing (polymerization and conversion into final products); (iii) Use Phase; and (iv) End-of-Life (EoL) Management [124]. The EoL stage represents a critical branch point with multiple pathways including recycling (mechanical/chemical), incineration (with or without energy recovery), landfilling, composting (for certain biopolymers), and, critically, loss to the environment. The relative prevalence and impact of these pathways differ significantly by polymer type (e.g., PET vs. LDPE), product design, and local waste management infrastructure. These interconnected stages are summarized in Figure 4.

This holistic approach enables the identification of environmental hotspots, such as greenhouse gas emissions during production or toxicity potentials from additives and facilitates comparative analysis between different product alternatives or waste management strategies [124].

#### Gaps and Advancements in LCA for Plastic Pollution

The current application of LCA methodology reveals significant gaps in addressing plastic pollution impacts. Traditional LCA frameworks have not fully integrated into the environmental effects of plastic pollution, particularly concerning microplastic emissions, which primarily occur during the use phase (e.g., abrasion, washing) and from mismanaged EoL waste and their subsequent ecosystem impacts. This represents a critical limitation given the escalating rates of plastic production and the global presence of microplastics. Consequently, significant efforts are now underway to develop robust characterization factors that can effectively incorporate microplastic emissions and their associated environmental impacts into standardized LCA frameworks [127,128]. These advancements are essential for creating comprehensive sustainability assessments that accurately reflect the true environmental costs of plastic products and technologies, thereby informing better policy and design decisions [129].

## 9. Advanced Adsorbent Materials for Targeted Removal

The development of advanced adsorbent materials represents a promising frontier for the targeted capture and removal of MPs, particularly suitable for polishing effluents or treating point-source pollution. Unlike conventional unit operations, these materials are designed at the molecular and nanoscale to maximize specific surface area, introduce tailored surface chemistries, and enable regeneration. However, their practical deployment hinges on key performance metrics: high sorption capacity, selectivity for MPs in complex aqueous matrices, and long-term stability or reusability. The following sections critically evaluate emerging adsorbent classes against these criteria, with key comparative data summarized in Table 7.

### 9.1. Engineered Sponges for Microplastics Removal

Engineered sponges, typically based on biopolymers like chitin or composite structures, represent an emerging technology for targeted MP removal. Their highly porous, compressible, and often hydrophobic structures facilitate the physical trapping and adhesion of microplastic particles [130,131]. For instance, chitin/graphene oxide (ChGO) composite sponges have demonstrated adsorption capacities for polystyrene (PS) MPs ranging from 5.90 to 8.46 mg/g, increasing with temperature from 25 to 45 °C [132]. They also show promising reusability, maintaining 72–90% removal efficiency for various PS types after three adsorption-desorption cycles [132]. The primary removal mechanism involves hydrophobic, electrostatic, and π–π interactions, which raise important selectivity challenges; these sponges may also non-specifically adsorb natural organic matter present in real water. Therefore, their most viable application may be as a reusable filter medium for controlled industrial effluents or as a tertiary polishing step where feedwater pre-treatment reduces fouling competitors.

**Table 7:** Comparative performance metrics of advanced adsorbents for microplastic removal.

Material Type	Max. Adsorption Capacity (mg/g)	Key MPs Tested	Reusability/Cycles	Key Advantages	Primary Challenges
Chitin/GO Sponge [132]	5.9–8.5	Polystyrene (PS)	72–90% efficiency after 3 cycles	Compressible, reusable, good for varied PS surface groups	Low capacity, selectivity issues in complex water
3D G@LDO [133]	209.4	Polystyrene (PS)	manuscript	High capacity, pH-stable, tunable chemistry	Cost, scalability, and selectivity data in real matrices
Magnetic CNTs [134]	1100–1650	PE, PET, Polyamide	~80% efficiency after 4 cycles	Very high capacity, magnetic separation enables easy recovery	Potential ecotoxicity, cost, long-term stability
Biochar [135,136]	>200 (general)	Various/Polystyrene (PS)	Varies; often considered low-cost disposable	Very low cost, sustainable feedstock, high surface area	Performance variability, spent material management

### 9.2. Graphene-Based Filters for Microplastics Removal

Graphene and its derivatives are attractive for advanced filtration due to their exceptional surface area, mechanical strength, and tunable surface chemistry [137]. Functionalized assemblies, such as 3D graphene-like carbon-assembled layered double oxides (G-LDO), show high capacity, removing PS MPs with a maximum adsorption of 209.39 mg/g and maintaining ≥80% efficiency across a broad pH range (3–11) [133]. A significant operational advantage is facilitated separation; magnetic carbon nanotubes (M-CNTs) achieve exceptional capacities (1100–1650 mg/g for polyethylene, PET, and polyamide) and near-complete removal, allowing for magnetic recovery of the sorbent-loaded MPs while retaining ~80% efficiency after four cycles [134]. While these materials exhibit very high capacity and easy recovery, their selectivity in environmentally relevant conditions, long-term stability, and potential ecotoxicity. While graphene-based materials are effective for MP removal, concerns about their own ecotoxicity persist. Most studies indicate low acute toxicity for well-prepared graphene filters, especially when immobilized in composites or sponges, and some demonstrate good biocompatibility and biodegradability. Nevertheless, the potential for nanomaterial leaching, long-term environmental accumulation, and chronic toxicity to aquatic organisms requires further investigation, particularly under real-world conditions [138].

### 9.3. Biochar-Based Filters for Microplastics Removal

Biochar, a carbon-rich porous material produced from biomass pyrolysis, has emerged as a particularly promising sustainable adsorbent. Its advantages include low cost, renewable feedstock, and highly tunable surface properties [135,138]. Activation can produce specific surface areas exceeding 2000 m^2^/g, contributing to high adsorption capacities, with reports of >200 mg/g for various MPs and >94.8% removal efficiency for PS MPs using modified biochar [135,136]. Pilot-scale studies suggest effectiveness in wastewater and surface water applications [135]. As a low-cost material, absolute selectivity is less critical than for engineered nanomaterials; biochar can be deployed as a disposable or regenerable filter media in decentralized systems, stormwater ponds, or as an additive in constructed wetlands. Its key challenges involve standardizing production for consistent performance and managing the spent material, though its potential for subsequent use as a soil amendment (if not contaminated with toxins) adds to its lifecycle appeal.

## 10. Conclusions and Future Perspectives

**Figure 5:**
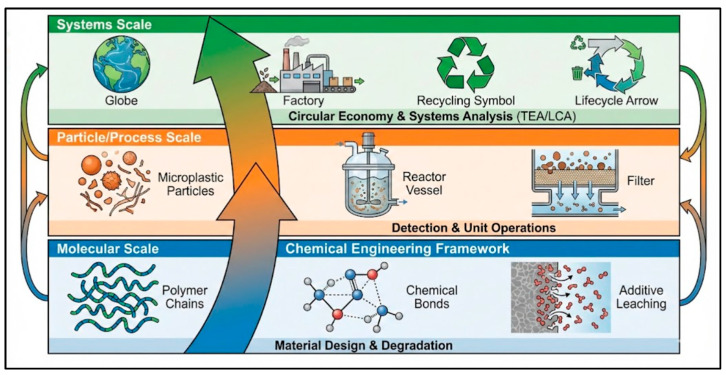
The Multi-Scale Chemical Engineering Framework.

As this review has demonstrated, the microplastic pollution challenge can be effectively framed across three interconnected scales where chemical engineering principles are paramount (Figure 5).

At the molecular and materials scale, the inherent properties of polymers dictate their environmental persistence and degradation pathways. The particle and process scale encompasses the detection, characterization, and physical/chemical removal of microplastics through engineered unit operations. Finally, the systems and societal scale require the application of techno-economic analysis, life cycle assessment, and circular economy models to evaluate the sustainability and viability of mitigation strategies. This multi-scale framework underscores the central thesis of this review: that addressing microplastic pollution effectively requires seamlessly linking fundamental science with scalable process engineering and holistic systems analysis.

Aligned with this framework, the review has analyzed the MP crisis through a chemical engineering lens, examining environmental transformations, evaluating mitigation technologies, and applying systems-level sustainability tools. The analysis confirms that MPs represent a complex, multi-scale challenge where polymer properties dictate environmental fate and where effective solutions require integration across molecular design, process engineering, and system optimization.

Key findings reveal that the formation and weathering of MPs are governed by reaction engineering principles, such as photo-oxidation and mechanical stress, which critically alter their surface properties and interactions. These changes underpin the environmental and toxicological risks of MPs, including their role as vectors for contaminants, underscoring the urgency of the challenge. Regarding mitigation, conventional wastewater treatment processes are necessary but are insufficient barriers, especially for nano-plastics. While advanced adsorbent materials including engineered sponges, graphene-based filters, and biochar demonstrate promising potential for targeted removal, their translation from the lab is contingent upon overcoming significant hurdles in scalability, cost, and long-term performance in complex matrices. Furthermore, techno-economic analysis (TEA) and life-cycle assessment (LCA) have emerged as critical, yet underdeveloped, tools for evaluating the true costs and sustainability of mitigation strategies; current methodologies must evolve to fully incorporate the impacts of MP emissions themselves.

Looking forward, addressing the MP crisis necessitates advances that bridge the gap between innovative materials and practical systems. The translation of advanced adsorbents from lab to scale requires focused efforts on cost-effective production, stability in real water matrices, efficient regeneration, and sustainable management of spent material. Future research must prioritize lifecycle assessments and pilot-scale integration to evaluate practical viability within circular economic frameworks. Ultimately, the path forward lies in developing hybrid systems that combine robust physical removal with destructive degradation, coupled with a fundamental shift toward polymers designed for circularity from the outset. The establishment of standardized analytical methods and LCA frameworks that include MP impacts is also a prerequisite for effective policy and regulation.

In sum, mitigating global microplastic pollution demands the core strengths of chemical engineering, the ability to link molecular-scale properties to process-scale operations and system-wide sustainability to forge the integrated pathways needed to transition from pollution to solution.

## Figures and Tables

**Table 1:** Typical Properties of Common Polymers Relevant to Environmental Interactions.

Polymer	Density (g/cm^3^)	Typical Zeta Potential (ζ, mV)	Key Functional Groups/Chemical Features	Predominant Environmental Interaction	Ref.
PE (Polyethylene)	0.91–0.96	−30 to −50	Aliphatic hydrocarbon	Hydrophobic sorption of PAHs, low surface reactivity	[1,2]
PP (Polypropylene)	0.90–0.92	−25 to −45	Aliphatic hydrocarbon, methyl side groups	Hydrophobic sorption, low surface reactivity	[1,2]
PS (Polystyrene)	1.04–1.07	−30 to −50	Aromatic ring	Hydrophobic sorption, π–π interactions with organics	[1,2]
PET (Polyethylene terephthalate)	1.34–1.40	−40 to −60	Aromatic ring, ester groups	Hydrogen bonding, sorption of metals/organics	[1,2]
PVC (Polyvinyl chloride)	1.30–1.45	−20 to −40	Aliphatic hydrocarbon, chloride	Hydrophobic and electrostatic interactions, sorption of metals	[1,2]
PA (Nylon)	1.13–1.15	−20 to −40	Amide groups (–CONH–)	Hydrogen bonding, sorption of metals and organics	[1,2,3]
PES (Polyether sulfone)	1.37–1.40	−40 to −60	Aromatic ring, sulfone, ether	Hydrogen bonding, sorption of polar organics/metals	[1,3]

Notes: (i) Zeta potential values are approximate and can vary with pH, ionic strength, and weathering. (ii) Environmental interactions may change after weathering or surface modification. (iii) Densities are for virgin polymers; additives and fillers can alter these values.

**Table 2:** Summary of primary and secondary microplastic sources.

Source Category	Example Products	Dominant Polymer Types	Classification	Key Release Mechanism	References
**Synthetic Textiles**	Clothing, carpets, fishing nets	PES (PET), PP, PA	Primary	Abrasion during washing and wearing	[5]
**Vehicle Tires**	Car, truck tires	Synthetic rubber, Styrene-Butadiene	Primary	Abrasion against road surfaces	[6]
**Road Markings**	Traffic paints, road signs	Acrylics, Epoxy resins	Primary	Abrasion from vehicle traffic	[7]
**Marine Coatings**	Ship hull paints	Polyurethane, Epoxy, Vinyl	Primary	Weathering, scraping, maintenance	[8]
**City Dust**	Artificial turf, paint flakes	Various	Primary	Abrasion, weathering, and wear	[9]
**Agricultural Products**	Mulch films, fertilizer coats	LDPE, PLA, PBAT	Primary	Photodegradation, soil abrasion	[10]
**Plastic Fragmentation**	Bottles, bags, packaging	PE, PP, PS, PET, PVC	Secondary	UV degradation & mechanical weathering	[12]
**Fishing Gear**	Discarded nets, ropes	PA, PP, PE	Secondary	Photodegradation and abrasion	[13]

**Note:** This table employs the release-based classification framework, where ‘Primary’ sources emit microplastics directly during a product’s use phase (e.g., abrasion, weathering). ‘Secondary’ sources result from the environmental fragmentation of larger plastic waste. For example, synthetic textile fibers are classified as primary because they are shed during washing/wearing, not from the breakdown of discarded clothing in the environment.

**Table 3:** Representative degradation rate data for common polymers under different environmental triggers.

Polymer	Trigger/Condition	Reported Metric	Approx. Rate Constant (k) or Half-Life (t_1/2_) *	Key Notes	Ref.
Polyethylene (PE) nanoparticles	UV radiation with TiO_2_ catalyst	Degradation rate constant	k = 2.6 × 10^−7^ h^−1^ (t_1/2_ ≈ 300 years) *	Photocatalytic degradation.	[17]
LDPE	Microbial consortia (e.g., *Pantoea* sp.)	Weight loss	~81% in 120 days (t_1/2_ ≈ 40 days) *	Biodegradation in controlled conditions.	[19]
PP	Microbial consortia (e.g., *Aneurinibacillus* spp.)	Weight loss	~37–46% in 140 days (t_1/2_ ≈ 140–180 days) *	Biodegradation rate depends on consortium.	[19]
Polypropylene (PP)	Simulated solar radiation (UV)	Fragmentation time constant	k (rel.) ~ 6× (relative to PS)	High fragmentation rate; ~100,000 daughter particles per mother particle.	[21]
Polystyrene (PS)	Simulated solar radiation (UV)	Fragmentation time constant	k (rel.) ~ 1× (baseline)	Used as baseline for comparison with PP.	[21]
LDPE	Simulated solar radiation (UV)	Fragmentation time constant	k (rel.) ~ 1.5× (relative to PS)	Degrades slower than PP but faster than PS.	[21]

* Calculated half-lives (t_1/2_) are approximate, assuming first-order kinetics for illustrative comparison. Actual degradation is often multi-phase and condition dependent.

**Table 4:** Comparison of analytical techniques for microplastic characterization; data synthesized from [96] (Huang et al., 2023) and [97] (Woo et al., 2021).

Technique	Principle	Size Range	Typical Limit of Detection (LOD)	Key Advantages	Key Limitations
Optical Microscopy	Light reflection	>100 µm	Visual: ~1–10 µm (depends on optics)	Low cost, simple, rapid visual sorting.	No chemical identification, subjective, prone to error.
SEM/TEM	Electron interaction	1 nm–500 µm	Imaging: <1 nm Elemental (EDS): ~1 µm	Exceptional spatial resolution, detailed morphology, elemental analysis (EDS).	High cost, requires expertise and vacuum, sample preparation can be complex.
µ-FTIR	Molecular vibrations	20 µm–500 µm	Spectral ID: ~10–20 µm	Provides polymer chemical identification, non-destructive, extensive spectral libraries.	Time-consuming for mapping, sensitive to water interference, requires particle isolation.
µ-Raman	Inelastic light scattering	1 µm–500 µm	Spectral ID: ~1 µm	High spatial resolution (<1 µm), minimal sample prep, works with aqueous samples.	Susceptible to fluorescence interference (can mask signal), laser can degrade some polymers.
Py-GC/MS	Thermal decomposition & mass spectrometry	All sizes	Mass-based: ~1 µg	Provides polymer mass quantification, highly sensitive and specific, identifies additives.	Destructive, complex data analysis, does not provide particle size or shape information.
TGA	Mass loss vs. temperature	All sizes	Mass-based: ~1 µg	Quantitative, high-throughput, good for mass concentration in complex samples.	Destructive, no particle counts, shape, or chemical ID of individual particles.
Near-Infrared (NIR) Spectroscopy	Absorption of NIR light by molecular overtone and combination vibrations.	>50 µm	Spectral ID: ~50–100 µm	Rapid, non-destructive, minimal sample prep, suitable for high-throughput sorting.	Lower spatial resolution than FTIR/Raman, requires calibration models, water interference.
Nano-thermal Analysis (AFM-Thermal)	Combines AFM with a nanoscale thermal probe to map thermal properties (e.g., Tg).	<100 nm (local property mapping)	Thermal transition detection at ~100 nm resolution.	Provides nanoscale thermal mapping (e.g., glass transition), correlates topology with material behavior.	Very slow, highly specialized, not for bulk analysis, requires flat samples.
X-Ray Diffraction (XRD)	Diffraction of X-rays by crystalline planes in a material.	All sizes (crystallite analysis)	Crystallite size: ~1–100 nm	Identifies crystalline phases, can differentiate polymer types (e.g., PE vs. PP), non-destructive.	Limited to crystalline/semi-crystalline polymers, not for amorphous plastics, requires sample preparation.
Atmospheric Solid Analysis Probe Mass Spectrometry (ASAP-MS)	Thermal desorption/ionization of solids under ambient conditions coupled with MS.	Single particles (µm to mm)	Mass-based: single-particle	Rapid analysis of single particles, minimal sample prep, generates polymer-specific mass spectra.	Semi-quantitative requires interpretation of complex spectra, instrument cost.

**Table 5:** Microplastic removal efficiencies of key wastewater treatment unit operations. The term ‘unit operations’ refers to discrete, standardized physical or chemical process steps, a fundamental chemical engineering concept used here to systematically evaluate and compare removal technologies based on their mechanistic function and scalability.

Treatment Process	Target Size Range	Reported Removal Efficiency	Key Removal Mechanism	References
Preliminary (Screening)	>6 mm	Variable (size-dependent)	Physical sieving	[100,101]
Primary Sedimentation	>100 µm	50–98%	Density separation (settling, flotation)	[100]
Activated Sludge	>10 µm	70–98%	Bio-flocculation and encapsulation	[100]
Membrane Bioreactor (MBR)	>0.1–0.4 µm	99.4–99.9%	Physical filtration and biological treatment	[102]
Dissolved Air Flotation (DAF)	10 µm–1 mm	85–95%	Bubble adhesion and flotation	[102]
Rapid Sand Filtration	>10 µm	>50% (of influent)	Depth filtration and adsorption	[102]
Electrocoagulation	Wide range	>90%	Charge neutralization and aggregation	[102]

**Table 6:** Comparative analysis of mitigation strategies: from treatment to redesign.

Strategy	Mechanism	Effectiveness	Scalability	Key Challenge	References
Membrane Bioreactors	Physical filtration & biodegradation	Very High	High (municipal)	Membrane fouling, high capital cost	[102]
Advanced Oxidation	Chemical mineralization	High (for MPs)	Medium	Energy-intensive, byproduct formation	[103]
Adsorbents (e.g., Biochar)	Surface adhesion & entrapment	Medium-High	Medium	Regeneration, disposal of spent media	[117]
Chemical Recycling	Depolymerization to monomers	High (purity dependent)	Growing	Requires sorted, clean plastic streams	[114,115]
Biodegradable Polymers	Microbial assimilation	Context-dependent	High	Controlled disposal infrastructure needed	[111,113]
Source Reduction	Prevent generation	Ultimate Solution	Varies	Consumer behavior, economic incentives	-

## Data Availability

No new data were created or analyzed in this study. Data sharing is not applicable to this article.
